# Treatment With Ruxolitinib and TAK‐779 Enhances GII.17 Human Norovirus Replication and Enables Serial Passaging in Human Intestinal Enteroids

**DOI:** 10.1111/gtc.70139

**Published:** 2026-08-01

**Authors:** Eri Hiraishi, Tsuyoshi Hayashi, Yoshiki Fujii, Hirokazu Kimura, Kosuke Murakami

**Affiliations:** ^1^ Department of Diagnostic Testing and Technology Research National Institute of Infectious Diseases, Japan Institute for Health Security Tokyo Japan; ^2^ Department of Health Science Gunma Paz University Graduate School of Health Sciences Takasaki Gunma Japan; ^3^ Department of Virology II National Institute of Infectious Diseases, Japan Institute for Health Security Tokyo Japan

**Keywords:** CXCR3/CCR5/CCR2 antagonist, human intestinal enteroids, human norovirus, JAK1/JAK2 inhibitor, serial passaging

## Abstract

Human norovirus (HuNoV) is a major cause of acute viral gastroenteritis. Although it can be replicated in vitro using human intestinal enteroids (HIEs), generating high‐titer viral laboratory stocks remains challenging due to low infection and passage efficiencies. Interferon response and chemokine signaling may be key host pathways that restrict efficient HuNoV replication. Here, we evaluated the effect of their inhibitors, ruxolitinib—a Janus kinase (JAK) 1/JAK2 inhibitor—and TAK‐779—a C‐X‐C motif chemokine receptor 3 (CXCR3)/C‐C motif chemokine receptor (CCR) 5/CCR2 antagonist—on HuNoV replication in wild‐type jejunal HIEs. TAK‐779 increased GII.17 HuNoV replication by 2.3‐ and 6.0‐fold at 48 and 96 h postinfection (hpi), respectively, compared with the DMSO‐treated control. In contrast, ruxolitinib alone did not affect viral replication. Notably, simultaneous treatment with these compounds further enhanced GII.17 replication by 6.0‐ and 10.7‐fold at 48 and 96 hpi, respectively, which enabled serial passaging. These findings imply that the simultaneous suppression of these pathways potentially benefits GII.17 HuNoV replication.

## Graphical abstract

We aimed to establish a simple method for GII.17 human norovirus (HuNoV) replication and serial passaging using wild‐type J2 human intestinal enteroids (HIEs). Treatment with ruxolitinib and TAK‐779, which target interferon and chemokine signaling, respectively, enhances viral replication and enables serial passaging of GII.17.

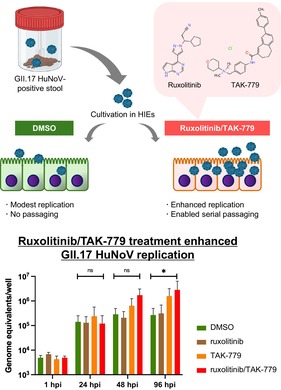

## 1 Introduction

Globally, human norovirus (HuNoV) causes acute viral gastroenteritis (World Health Organization 2015). Norovirus belongs to the family Caliciviridae and is classified into 10 genogroups: GI–GX (Chhabra et al. 2019). GI, GII, GIV, GVIII, and GIX infect humans, with GII being the main cause of norovirus outbreaks (Tran et al. 2013; Van Beek et al. 2018). HuNoV infection spreads easily via human–human or human–object–human contact (Lopman et al. 2012). Among the genotypes capable of infecting humans, GII.4 is the predominant cause of outbreaks worldwide, whereas GII.17 causes disease sporadically (Chan et al. 2017). However, during the 2024–2025 season, GII.17 cases increased markedly, unexpectedly exceeding those of GII.4 (Barclay and Vinjé 2025).

For many years, no reproducible culture system has been capable of supporting HuNoV replication and passaging. However, several culture systems, such as in vitro tissue stem cell‐derived human intestinal enteroids (HIEs) and zebrafish larvae/embryos, have recently been developed (Ettayebi et al. 2016; Tan et al. 2021; Van Dycke et al. 2019). Among these, HIEs are the most widely employed in vitro HuNoV replication models in laboratories to study HuNoV biology, including infection mechanisms and viral inactivation (Ayyar et al. 2023; Costantini et al. 2018; Escudero‐Abarca et al. 2022; Hosmillo et al. 2020). However, the ability to produce high‐titer stocks is limited by low infection and passaging efficiencies, delaying research progress (Ettayebi et al. 2016).

A plausible explanation for the low replication efficiency is the existence of host restriction factors preventing efficient replication and passaging of HuNoVs in HIEs. The interferon (IFN)‐mediated antiviral response counteracts many viral infections and is activated upon HuNoV infection in HIEs (Hosmillo et al. 2020; Kaur et al. 2026; Lin et al. 2020). Importantly, inhibition of the Janus kinase (JAK)/signal transducer and activator of transcription (STAT) signaling pathway, a key regulator of IFN response, such as by ruxolitinib or genetic modification, enhances HuNoV replication in HIEs, demonstrating the involvement of an IFN response in anti‐HuNoV activity (Hosmillo et al. 2020; Lin et al. 2020; Mirabelli et al. 2022).

Recently, Kaur et al. (2026) demonstrated that specific chemokine‐based signaling likely acts as another host restriction factor limiting adequate HuNoV replication and passaging in HIEs. In addition, GII.3 HuNoV infection induced the expression of several chemokines, such as C‐X‐C motif chemokine ligand (*CXCL*)10 and *CXCL11*, in HIEs. Interestingly, these two genes were also induced in the genetically modified HIEs J2 STAT1‐KO and J4FUT2‐KI (non‐secretor HIEs overexpressing *FUT2*), both with reduced transcription related to IFN response. These results suggest that such chemokine signaling was independent of IFN signaling. In a genetically modified HIE (J4FUT2‐KI), the inhibition of chemokine signaling by TAK‐779 (C‐X‐C motif chemokine receptor 3 [CXCR3]/C‐C motif chemokine receptor [CCR] 5/CCR2 antagonist) markedly enhanced GII.3 HuNoV replication and supported sustained passaging.

Interestingly, TAK‐779 also enhanced GII.17 and GI.1 replication in HIEs, but the effect was less evident compared with GII.3 (Kaur et al. 2026). However, TAK‐779 did not affect GII.4 replication in HIEs, suggesting the need for further optimization in other genotypes. Moreover, passaging experiments were performed using genetically modified HIEs, that is, J4FUT2‐KI, which allow the replication of multiple HuNoV genotypes (Ettayebi et al. 2024). In this study, we focused on wild‐type HIEs rather than genetically modified lines, aiming to establish a simpler and more broadly applicable system for HuNoV replication and serial passaging, particularly for GII.17.

## 2 Results

### 2.1 Effect of Ruxolitinib and TAK‐779 on HuNoV Replication in HIEs

**FIGURE 1:**
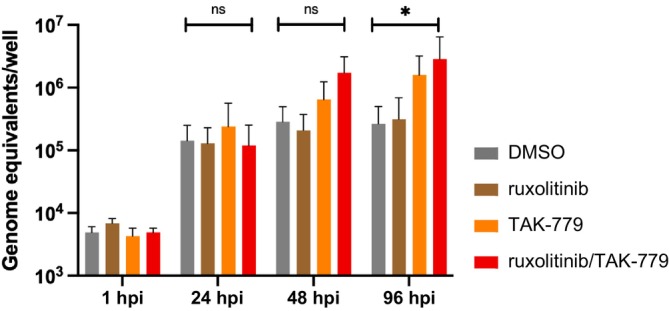
Treatment with ruxolitinib and TAK‐779 enhances GII.17 replication in HIEs. J2 monolayers were pretreated with ruxolitinib (5 μM) and/or TAK‐779 (30 μM) for 3 h and then inoculated with a stool filtrate containing GII.17 (1.6 × 10^5^ GEs/well) in a differentiation medium supplemented with the corresponding compounds and 500 μM GCDCA for 1 h at 37°C. After washing, the cells were cultured with the specified compounds and GCDCA. The cells and culture supernatants were collected at 24, 48, and 96 hpi, and viral RNA was quantified by RT‐qPCR. Differentiation medium containing DMSO and 500 μM GCDCA served as the vehicle control. Data are presented as the mean ± SD (*n* ≥ 5). Statistical significance was assessed using the Kruskal–Wallis test followed by Steel's multiple‐comparison test. Asterisks indicate statistically significant differences compared with DMSO‐treated controls at each time point (*p < 0.05). ns, not significant; hpi, h postinfection.

Previous studies strongly suggest that the IFN response mediated by the JAK/STAT pathway and chemokine signaling are two key host restriction factors preventing efficient HuNoV replication and continuous passaging of certain HuNoV genotypes, such as GII.3 and GII.17, in HIEs (Hosmillo et al. 2020; Kaur et al. 2026; Lin et al. 2020). Therefore, we first investigated whether simultaneous exposure to these two compounds inhibiting such signaling pathways enhanced HuNoV replication, allowing serial passaging in nongenetically modified (i.e., wild‐type) HIEs. We evaluated the effects of their inhibitors, ruxolitinib (JAK1/JAK2 inhibitor) and TAK‐779 (CXCR3/CCR5/CCR2 antagonist), on viral replication in J2 HIEs, widely used for HuNoV culture. J2 monolayers were pretreated with ruxolitinib and/or TAK‐779 for 3 h, and infected with GII.17 HuNoV in the presence of corresponding inhibitors at concentrations selected based on earlier reports (Hosmillo et al. 2020; Kaur et al. 2026). At 24, 48, and 96 h postinfection (hpi), viral replication was evaluated by reverse transcription‐quantitative polymerase chain reaction (RT‐qPCR) using specific primers. Although ruxolitinib blocked the induction of several interferon‐stimulated genes (ISGs; e.g., *RSAD2* and *ISG56*) in virus‐infected HIEs (Figure S1), it did not affect viral replication compared with the DMSO‐treated control at any time point (Figure 1). TAK‐779 increased viral replication at 48 and 96 hpi by 2.3‐ and 6.0‐fold, respectively, but these differences were not statistically significant (Figure 1). In contrast, treatment with ruxolitinib and TAK‐779 increased HuNoV RNA copies compared with the control at 48 and 96 hpi (6.0‐ and 10.7‐fold increase, respectively), whereas the difference from the DMSO control was statistically significant only at 96 hpi (*p* < 0.05).

**FIGURE 2:**
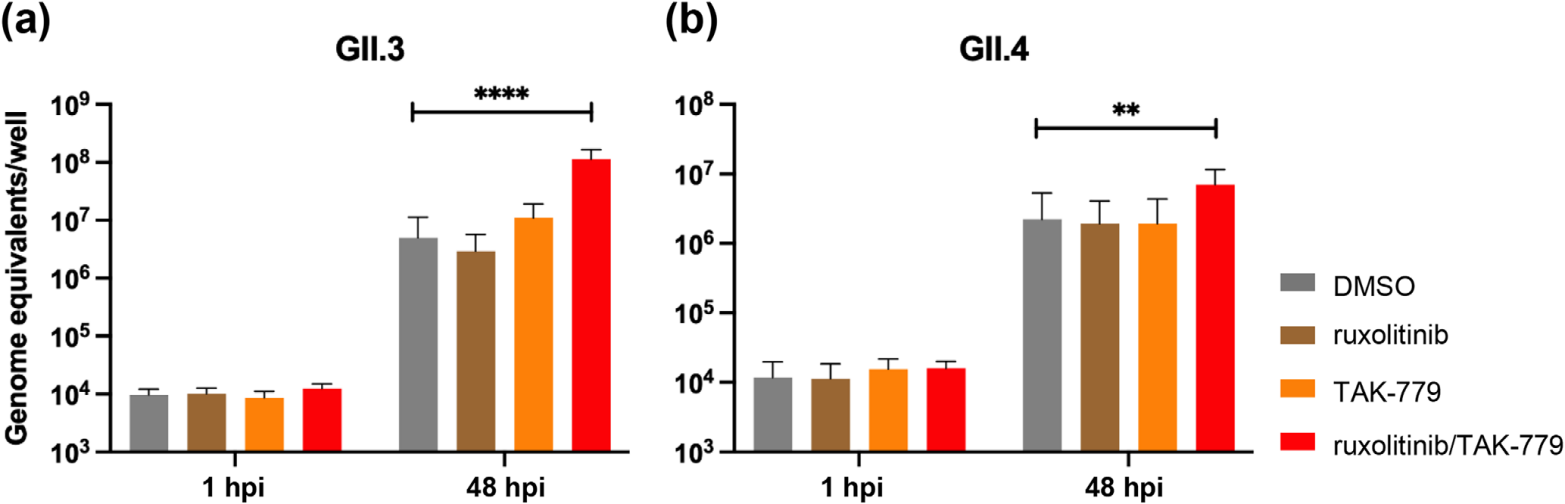
Effects of ruxolitinib and TAK‐779 on GII.3 and GII.4 HuNoV replication in HIEs. J2 monolayers pretreated with ruxolitinib (5 μM) and/or TAK‐779 (30 μM) were infected with stool filtrate containing GII.3 (4.3 × 10^5^ GEs/well) (a) or GII.4 (1.1 × 10^7^ GEs/well) (b), as shown in Figure 1. At 48 hpi, the cells and culture supernatants were collected, and viral RNA was quantified by RT‐qPCR. Data are presented as the mean ± SD (*n* ≥ 9). Statistical significance was assessed using the Kruskal–Wallis test followed by Steel's multiple‐comparison test. Asterisks indicate statistically significant differences compared with DMSO‐treated controls (**p < 0.01, ****p < 0.0001). hpi, h postinfection.

Similar to the GII.17 infection results (Figure 1), ruxolitinib alone showed no significant difference in GII.3 replication compared with the DMSO‐treated control (Figure 2a). In contrast, TAK‐779 alone increased GII.3 HuNoV RNA copy number by 2.2‐fold compared with the control at 48 hpi. Ruxolitinib/TAK‐779 resulted in a 22.7‐fold increase (*p* < 0.0001, Figure 2a). Furthermore, ruxolitinib or TAK‐779 alone did not affect GII.4 HuNoV replication, but both together increased GII.4 replication by 3.1‐fold (*p* < 0.01; Figure 2b). These data indicated that treatment with ruxolitinib and TAK‐779 enhanced HuNoV replication in GII.17, GII.3, and GII.4.

### 2.2 Treatment With Ruxolitinib and TAK‐779 Enabled GII.17 HuNoV Passaging

Next, we evaluated whether exposure to both ruxolitinib and TAK‐779 enabled serial passaging of GII.17 in HIEs. The infectious progeny of human sapoviruses (Caliciviridae) are retained more within the cells compared with the culture supernatant (Fukuda et al. 2025). Therefore, we first evaluated whether cell‐derived progeny were more infectious than supernatant‐derived viruses in HIEs. We prepared supernatant‐ and cell‐derived viruses—the passage 1 (P1) virus stock—as described in the Experimental Procedures and infected HIEs to compare their growth. Cell‐derived viruses replicated more robustly than supernatant‐derived ones between 3 and 96 hpi: 1222‐ and 783‐fold increase with cell‐derived vs. 118‐fold and no detectable increase with supernatant‐derived viruses at 2‐ and 10‐fold diluted stocks, respectively (Figure S2). Therefore, we employed cell‐derived viruses in subsequent passaging experiments.

**FIGURE 3:**
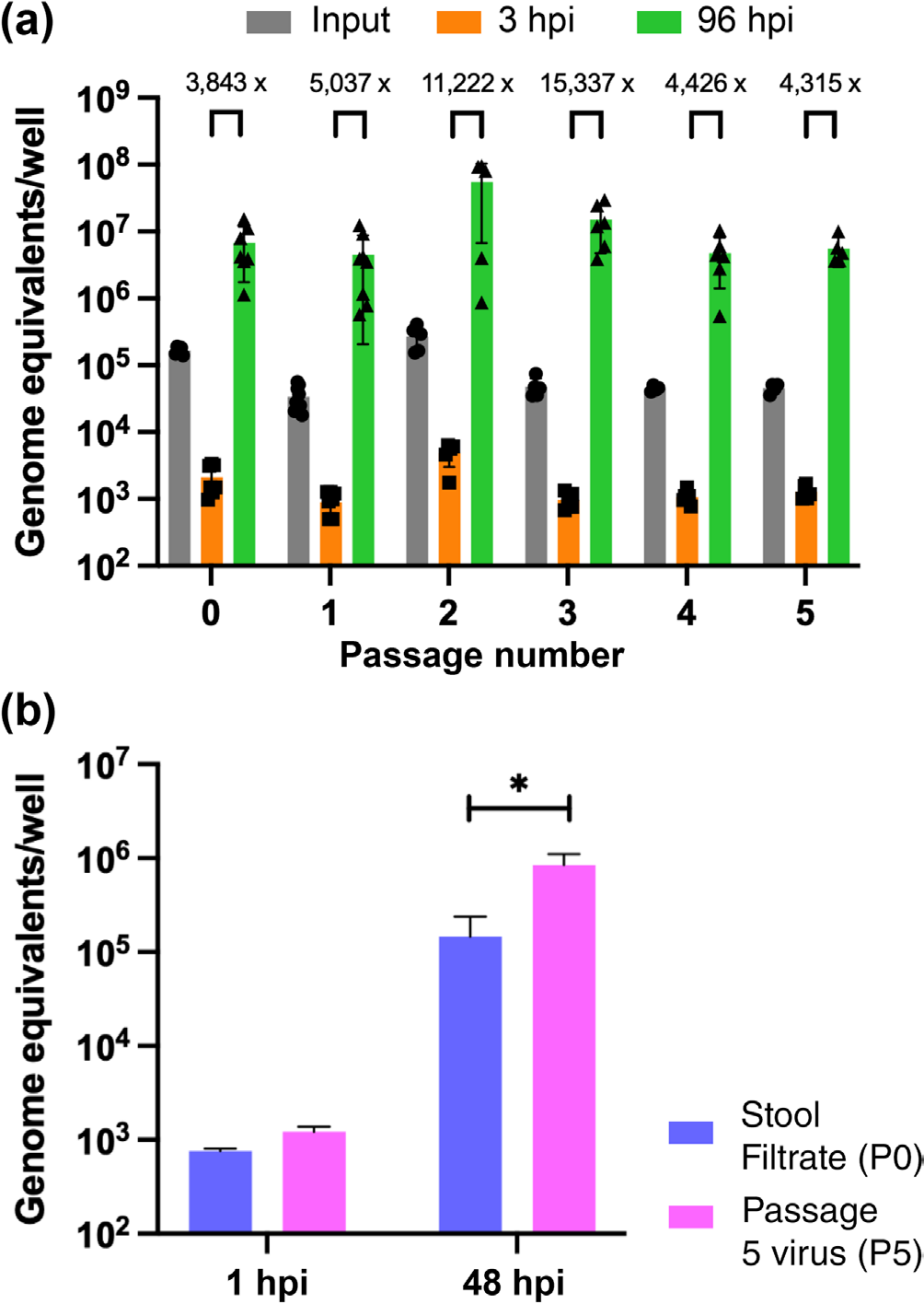
Treatment with ruxolitinib and TAK‐779 enables serial passaging of GII.17, and passaged viruses exhibit higher viral RNA copies than stool‐derived viruses in HIEs. (a) J2 monolayers pretreated with ruxolitinib (5 μM) and TAK‐779 (30 μM) were infected with stool filtrate containing GII.17 (1.6 × 10^5^ GEs/well) in a differentiation medium supplemented with the specified compounds and GCDCA for 3 h at 37°C. At 96 hpi, the cells were harvested, sonicated, and centrifuged. A portion of the clarified supernatants was used for RT‐qPCR quantification of viral RNA. For serial passaging, the virus was used to inoculate newly prepared J2 monolayers under the same conditions for up to five passages. Data are presented as mean ± SD (*n* ≥ 5). (b) J2 monolayers were inoculated with 4.6 × 10^4^ GEs per well of stool‐derived GII.17 or P5 virus stock in differentiation medium supplemented with GCDCA for 1 h at 37°C. Following infection, cells and culture supernatants were collected at 48 hpi, and viral RNA was quantified by RT‐qPCR. Data are presented as mean ± SD (*n* = 4). Statistical significance was assessed using the Welch *t*‐test. Asterisks indicate statistically significant differences compared with stool‐derived infection at 48 hpi (*p < 0.05). hpi, h postinfection.

The cell‐derived P1 virus stock was passaged onto fresh J2 HIEs in the presence of ruxolitinib and TAK‐779. Then the P2 stock was collected using the same procedure; serial passaging was continued up to P5. The number of HuNoV RNA copies at 96 hpi increased by 5037‐fold in P1, 11,222‐fold in P2, 15,337‐fold in P3, 4426‐fold in P4, and 4315‐fold in P5 compared with those at 3 hpi (Figure 3a). In P1 and subsequent passaging, HuNoV RNA copies at 96 hpi ([4.5 ± 4.3] × 10^6^–[5.5 ± 4.8] × 10^7^ genome equivalents (GEs)/well) exceeded the input number of HuNoV RNA copies ([3.4 ± 1.4] × 10^4^–[2.7 ± 1.1] × 10^5^ GEs/well), and virus yields remained stable (Figure 3a). To further investigate the role of ruxolitinib and TAK‐779 in serial passaging, P1 virus stocks generated under DMSO or ruxolitinib/TAK‐779 treatment conditions were normalized to equalize viral RNA copy numbers and were inoculated onto fresh J2 HIEs (Figure S3). P1 virus stocks generated under DMSO failed to support viral RNA replication with either DMSO or ruxolitinib/TAK‐779 treatment. In contrast, P1 virus stocks generated under ruxolitinib/TAK‐779 treatment supported viral RNA replication under both conditions, with RNA copy numbers increasing by 102‐ and 17,177‐fold under DMSO and ruxolitinib/TAK‐779 treatments, respectively.

Finally, we compared the viral replication of P0 (stool filtrate) and P5 GII.17 in J2 HIEs. J2 HIEs were inoculated with either stool filtrates or P5 virus (an equal number of HuNoV RNA copies) and then cultured for 48 h without ruxolitinib and TAK‐779. Viral replication was significantly higher with P5 virus than with the stool filtrates—stool filtrate‐infected: (1.5 ± 0.9) × 10^5^ GEs/well vs. P5 virus‐infected: (8.5 ± 2.5) × 10^5^ GEs/well at 48 hpi (*p* < 0.05; Figure 3b). These data indicated that passaged GII.17 could replicate in J2 HIEs, even without ruxolitinib and TAK‐779.

**FIGURE 4:**
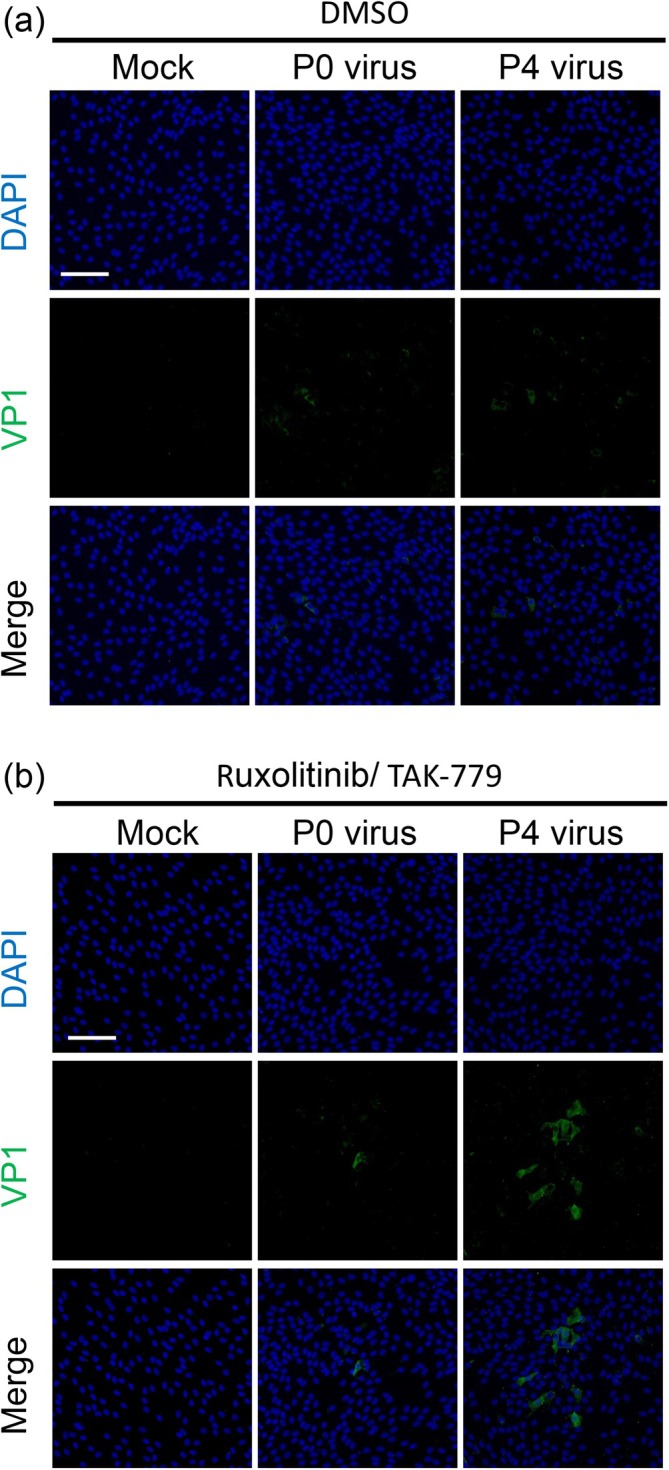
Detection of GII.17 VP1 in HIEs by immunofluorescence. J2 monolayers pretreated with DMSO (a) or ruxolitinib (5 μM) and TAK‐779 (30 μM) (b) were infected with mock inoculum (left panels), stool filtrate containing GII.17 (middle panels), or P4 virus stock (right panels) in the presence of DMSO or specified compounds and GCDCA. At 96 hpi, the cells were fixed with 4% paraformaldehyde and permeabilized with 1% Triton X‐100. GII.17‐positive cells were detected using GII.17‐specific antiserum (green) and stained with DAPI (blue). Scale bars: 100 μm. DAPI, 4′,6‐diamidino‐2‐phenylindole; VP1, viral protein 1.

To confirm that the serially passaged virus remained infectious, we assessed the expression of viral protein 1 (VP1), a structural protein in infected cells, using immunofluorescence. Representative images showed VP1‐positive cells postinfection with either P0 or P4 virus under DMSO and ruxolitinib/TAK‐779 treatments (Figure 4). This analysis was qualitative and did not compare infection efficiency between conditions.

## 3 Discussion

The inability of HuNoV culture systems to support indefinite passage and generate high‐titer viral stocks has been an obstacle to HuNoV research since its development in 2016. Recently, Kaur et al. (2026) reported that TAK‐779, an inhibitor of chemokine signaling, increased GII.3 HuNoV replication, enabling serial passage and the generation of viral stocks in genetically modified HIEs with reduced IFN response. This study reported that ruxolitinib and TAK‐779, which target IFN and chemokine signaling, respectively, significantly enhanced viral replication and supported the serial passaging of GII.17 in wild‐type HIEs, establishing a simpler method for serial passage.

GII.17 could be passaged until P8 using induced pluripotent stem cell (iPSC)‐derived intestinal epithelial cells (IECs). However, the possible generation of a virus stock has not been detailed (Sato et al. 2019). A more recent report suggested that GII.17 could be passaged using zebrafish embryos and that the virus retained infectivity in iPSC‐derived IECs (Kotaki et al. 2025). However, HuNoV culture in zebrafish may depend on access to a dedicated facility and personnel trained for embryo handling and microinjection. Collectively, these HIE‐ and zebrafish‐based strategies represent a major technical advancement in HuNoV studies that can support the development of antiviral agents and vaccines.

Our experiments demonstrated enhanced viral replication across passages until P5, with 4315–15,337‐fold increases (Figure 3a). P1 generated under DMSO did not support viral replication during the subsequent passage, unlike P1 generated under ruxolitinib/TAK‐779 treatment (Figure S3). These findings suggest that generating viral stocks with ruxolitinib and TAK‐779 is critical for subsequent passaging. Ruxolitinib and TAK‐779 may enhance viral RNA replication and facilitate the production of progeny capable of establishing subsequent rounds of infection. Further studies utilizing virus stocks generated under treatment with individual compounds can clarify the respective contributions of ruxolitinib and TAK‐779 to serial passaging. This study generated virus stocks from infected cells, whereas previous studies used stocks derived from a mixture of infected cells and culture supernatants (Kaur et al. 2026). As cell‐derived viruses showed greater replication ability than supernatant‐derived viruses in our experiments (Figure S2), they may provide a practical approach for HuNoV propagation in HIEs. Furthermore, viral replication was significantly greater for P5 GII.17 than for P0 (stool filtrate) in J2 HIEs (Figure 3b), which may be attributable either to viral adaptation to HIEs during serial passaging or to the suppressed influence of host‐derived components in stool samples, or to both. However, this study does not directly demonstrate whether viral adaptation occurs during passaging, for which further studies are required, including a comparative analysis of P0 and P5 sequences and characterization of recombinant viruses harboring the mutations identified.

Ruxolitinib alone did not affect HuNoV replication in J2 HIEs: 0.6‐, 0.9‐, and 0.7‐fold increase for GII.3, GII.4, and GII.17, respectively, compared with DMSO at 48 hpi. In contrast, TAK‐779 alone increased GII.3 and GII.17 replication by 2.2‐ and 2.3‐fold for GII.3 and GII.17, respectively, at 48 hpi, although these changes were not statistically significant. Treatment with ruxolitinib and TAK‐779 enhanced HuNoV replication, with statistically significant effects observed for GII.3 and GII.4 at 48 hpi and for GII.17 at 96 hpi: 22.7‐, 3.1‐, and 10.7‐fold increases, respectively (Figures 1 and 2). This pattern is consistent with a previous report of TAK‐779 markedly enhancing GII.3 replication in wild‐type and genetically modified HIEs, including J4 FUT2‐KI, which exhibited a reduced IFN response, although J4 FUT2‐KI supported more efficient viral growth (Kaur et al. 2026), implying that inhibition of both pathways may be necessary to maximize HuNoV replication in HIEs. However, whether this effect reflects a synergistic interaction between the two compounds remains unclear. In contrast to the IFN response to viral infection, the antiviral role of epithelial chemokines has been less extensively studied, and further investigations, such as characterization of chemokine gene knockout HIEs, are necessary.

Notably, TAK‐779 alone did not affect GII.4 replication in HIEs, whereas ruxolitinib and TAK‐779 enhanced GII.4 replication in HIEs: a 3.1‐fold increase at 48 hpi (Figure 2b). GII.4 infection does not induce measurable chemokine secretion in HIEs, unlike GII.3 infection (Kaur et al. 2026); consistent with this finding, TAK‐779 alone did not affect GII.4 replication (Figure 2b). During GII.4 infection, chemokine signaling does not appear to suppress viral replication, suggesting that GII.4 may utilize a mechanism distinct from that of other genotypes. The processes by which ruxolitinib and TAK‐779 promote viral replication remain unclear. One possibility is that GII.4 replication is restricted by multiple host immune responses rather than a single pathway, such that inhibition of an individual pathway is insufficient, whereas simultaneous suppression may induce an observable enhancement in viral replication. Further investigation is required to determine whether the effect is an outcome of synergism.

In our study, ruxolitinib did not affect viral replication, inconsistent with previous reports (Hosmillo et al. 2020; Mirabelli et al. 2022); the reason remains unclear. A possible explanation is that experimental conditions, including HuNoV genotypes, strains, and/or the multiplicity of infection, may influence the effect of ruxolitinib on HuNoV replication. Ruxolitinib significantly enhanced the replication of only one of three GII.4 strains in three‐dimensional (3D)‐HIEs compared with DMSO (Mirabelli et al. 2022). Another possibility is that a 3 h pretreatment followed by postinfection exposure to ruxolitinib may be insufficient to inhibit antiviral IFN responses to favor viral replication, although this treatment significantly suppressed the transcription of ISGs, such as *RSAD2* and *ISG56* (Figure S1). We chose this condition to maximize the proviral effect of TAK‐779 because Kaur et al. (2026) have reported that a 3‐h pretreatment followed by postinfection treatment with TAK‐779 markedly enhanced GII.3 replication compared with a 24 h pretreatment and postinfection exposure. Further optimization of treatment conditions, such as duration and/or concentrations of ruxolitinib and TAK‐779, may enhance their proviral effects.

This study evaluated the effects of ruxolitinib and TAK‐779 on HuNoV replication in an HIE from a single donor. Differences in donor background may affect susceptibility to HuNoV infection and responsiveness to these compounds. Therefore, the effect of ruxolitinib and TAK‐779 on HIE lines established from multiple donors and iPSC‐derived IECs may help improve HuNoV culture systems.

In conclusion, we demonstrated that simultaneous treatment with two compounds, ruxolitinib and TAK‐779, significantly enhanced HuNoV replication and enabled serial passaging of GII.17 up to P5 in J2 HIEs. These findings can contribute to developing a simplified HuNoV culture system that enables the generation of viral stocks from rare clinical isolates and reverse genetics‐derived viruses, as well as continuous passaging.

## 4 Experimental Procedures

### 4.1 HIE Culture

The jejunal HIE J2 line was obtained from Baylor College of Medicine (Houston, TX, USA) under a material transfer agreement and cultured as previously described (Ettayebi et al. 2016; Murakami et al. 2020). Briefly, 3D HIEs were embedded in Matrigel and cultured in complete medium containing growth factors (CMGF[+]) (Ettayebi et al. 2016) or IntestiCult Organoid Growth Medium (STEMCELL Technologies, Vancouver, Canada) for 5 days, with replacement every 2–3 days. To prepare two‐dimensional (2D) monolayers, 3D HIEs were treated with TrypLE Express Enzyme (Thermo Fisher Scientific, Waltham, MA, USA) at 37°C for 4 min. The cells were then centrifuged at 400 × *g* for 5 min at 4°C, resuspended in a 1:1 mixture of CMGF(+) and IntestiCult Organoid Growth Medium containing ROCK inhibitor Y‐27632 (10 μM; Sigma‐Aldrich, St. Louis, MO, USA), and seeded onto collagen IV‐coated 96‐well plates (1 × 10^5^ cells per well). After 2 days, the medium was replaced with a differentiation medium (Ettayebi et al. 2016), and the cells were cultured for another 3 days.

### 4.2 Stool Filtrates

GII.17[GII.P17] and GII.4[GII.P16] (EF24‐1 and EF23‐1; GenBank Accession Nos. LC919492.1 and LC777250.1, respectively) were used. HuNoVs were prepared from stool samples collected from HuNoV‐positive patients. The samples were mixed with phosphate‐buffered saline (PBS; Fujifilm Wako, Osaka, Japan) at a 1:9 ratio and vortexed thoroughly to obtain a 10% suspension. It was then centrifuged at 10,000 × *g* for 5 min at 4°C, and the supernatant was passed through a 0.22‐μm filter. Finally, the filtrates were aliquoted and stored at −80°C. GII.3[GII.P21] (TCH04‐577) HuNoV was prepared as previously described (Murakami et al. 2020).

### 4.3 HuNoV Infection

HuNoV was infected in the presence of ruxolitinib and/or TAK‐779 based on a previous report (Hayashi et al. 2021). Briefly, J2 monolayers were pretreated with either ruxolitinib (5 μM; T1829, TargetMol, Boston, MA, USA), TAK‐779 (30 μM; T7499, TargetMol), both, or DMSO (control) at 37°C under 5% CO_2_ for 3 h, and then inoculated with HuNoV filtrates—1.6 × 10^5^ GEs/well for GII.17, 1.1 × 10^7^ GEs/well for GII.4, and 4.3 × 10^5^ GEs/well for GII.3—in a differentiation medium supplemented with 500 μM sodium glycochenodeoxycholate (GCDCA; Sigma‐Aldrich) with DMSO or the indicated compounds at 37°C for 1 h. The monolayers were then washed twice with complete medium without growth factors (CMGF[−]). The cells were cultured in a differentiation medium supplemented with 500 μM GCDCA containing DMSO or the indicated compounds until the set time elapsed.

### 4.4 Passaging of HuNoV and Preparation of Its Stock

J2 monolayers pretreated with 5 μM ruxolitinib and 30 μM TAK‐779 were infected with GII.17 HuNoV for 3 h. At 96 hpi, the culture supernatants—100 μL per well—were harvested and centrifuged at 400 × *g* for 10 min at 4°C. The clarified supernatants were collected and stored at −80°C as the supernatant‐derived P1 virus stock. The cells were harvested, resuspended in 100 μL of differentiation medium, sonicated—3 cycles of 1 min on/1 min off—on ice, and centrifuged at 400 × *g* for 10 min at 4°C. The clarified supernatants were collected and stored at −80°C as the cell‐derived P1 virus stock. The supernatant‐ and cell‐derived stocks were diluted 2‐ or 10‐fold in differentiation medium and used to infect J2 HIEs as described above. As the cell‐derived viruses had a higher viral RNA copy number than supernatant‐derived viruses at 96 hpi (Figure S2), the cell‐derived stock was used for passaging experiments. A portion of the stock was used to quantify viral RNA by RT‐qPCR. For serial passaging, the virus stock was diluted in fresh differentiation medium containing 5 μM ruxolitinib, 30 μM TAK‐779, and 500 μM GCDCA and used to inoculate newly prepared J2 monolayers pretreated with the specified compounds. The GII.17 inoculum was prepared from the diluted virus stocks containing 3.4 × 10^4^–2.7 × 10^5^ GEs per well. The procedure was repeated five times to prepare virus stocks up to P5.

### 4.5 Quantification of the HuNoV Genome

At the indicated hpi, the infected cells and supernatants were collected for RNA extraction with the MagMAX CORE Nucleic Acid Purification Kit (Thermo Fisher Scientific) per the manufacturer's instructions. The extracted HuNoV RNA (5 μL) was quantified by RT‐qPCR (final reaction volume: 20 μL) using a Luna Universal Probe One‐Step RT‐qPCR Kit (New England Biolabs, Ipswich, MA, USA) on a CFX96 Deep Well Real‐Time PCR Detection System (Bio‐Rad, Hercules, CA, USA). The GII‐specific primer/probe sets employed were as described previously (Kageyama et al. 2003). A standard curve was generated using a plasmid containing the conserved ORF1–ORF2 junction of HuNoV GII, and viral GEs in each sample were quantified as described previously (Hayashi et al. 2021; Murakami et al. 2020).

### 4.6 RT‐qPCR to Measure ISG mRNA Expression

Total RNA was extracted from the infected cells and supernatant using a MagMAX CORE Nucleic Acid Purification Kit, followed by reverse transcription to synthesize cDNA using a PrimeScript RT Reagent Kit (Perfect Real Time; TAKARA, Shiga, Japan). The cDNA was diluted 10‐fold and used as the template. qPCR was performed using equal amounts of cDNA, primers specific for the target genes, and TB Green Premix Ex Taq II (Tli RNaseH Plus; TAKARA) on a QuantStudio 3 Real‐Time PCR System (Applied Biosystems, Thermo Fisher Scientific) with the following program—95°C for 30 s, followed by 40 cycles at 95°C for 5 s and 60°C for 34 s. *GAPDH* served as an endogenous control to normalize *ISG* expression. Primers used were as follows:—5′‐GGAGCGAGATCCCTCCAAAAT‐3′ (forward) and 5′‐GGCTGTTGTCATACTTCTCATGG‐3′ (reverse) for *GAPDH* (Ding et al. 2018); 5′‐AACCTCTGAGCATCCTGGTG‐3′ (forward) and 5′‐GAAGGTCAGCCAGAACAGGT‐3′ (reverse) for *ISG15*; 5′‐TGGGTGCTTACACCTGCTG‐3′ (forward) and 5′‐TGAAGTGATAGTTGACGCTGGT‐3′ (reverse) for *RSAD2* (Wu et al. 2018); and 5′‐TTGATGACGATGAAATGCCTGA‐3′ (forward) and 5′‐CAGGTCACCAGACTCCTCAC‐3′ (reverse) for *ISG56*.

### 4.7 Immunofluorescence to Detect GII.17 HuNoV VP1

J2 monolayers in 96‐well plates were fixed with 4% paraformaldehyde for 20 min at room temperature and permeabilized with 1% Triton X‐100 for 5 min at room temperature. The cells were then incubated in blocking solution—PBS containing 5% bovine serum albumin (BSA; Sigma‐Aldrich)—for 1 h at room temperature, and then overnight at 4°C with rabbit anti‐GII.17/Kawasaki308 virus‐like particles serum (1:500 in 5% BSA; a gift from Dr. Kazuhiko Katayama, Kitasato University, Japan). After washing the cells three times with PBS containing 0.1% Tween 20 (PBS‐T), they were incubated with goat anti‐rabbit Alexa Fluor 488 (Thermo Fisher Scientific) diluted 1:1000 in 5% BSA for 1 h at room temperature in the dark. Nuclei were stained with 4′,6‐diamidino‐2‐phenylindole (DAPI; DOJINDO, Kumamoto, Japan) at 1:1000 in PBS for 10 min at room temperature. Finally, the cells were washed three times with PBS‐T, followed by two washes with PBS. Immunofluorescence was detected with an APEXVIEW APX100 fluorescence microscope (Evident, Tokyo, Japan).

### 4.8 Statistical Analysis

Unless otherwise stated, experimental data were obtained from ≥ 2 independent experiments and presented as mean ± standard deviation (SD). Statistical analyses used GraphPad Prism 10 (GraphPad Software, San Diego, CA, USA). Statistical significance was assessed using the Welch *t*‐test for comparisons between two groups and the Kruskal–Wallis test followed by Steel's multiple‐comparison test for multiple‐group comparisons. A *p* value < 0.05 was considered statistically significant.

## Author Contributions

Conceptualization: E.H., T.H., and K.M.; Methodology: E.H., T.H., and K.M.; Validation: E.H. and T.H.; Formal Analysis: E.H., T.H., and K.M.; Investigation: E.H., Y.F., and T.H.; Writing – original draft: E.H. and T.H.; Supervision: H.K. and K.M.; Project administration: E.H., H.K., and K.M. All authors have read and agreed to the published version of the manuscript.

## Funding

This research was supported in part by the Japan Society for the Promotion of Science (KAKENHI Grant JP23K18381 to T.H. and K.M. and Grant JP25H00755 to T.H. and K.M.), the Japan Agency for Medical Research and Development (AMED) (Grant JP23fk0108669 to T.H. and Grant JP23fk0108667 to Y.F., H.K., and K.M.), and Health and Labour Sciences Research Grants from the Ministry of Health, Labour and Welfare of Japan (Grant Number JPMH25KA1001 to K.M.).

## Ethics Statement

This study was approved by the Ethics Committee of the National Institute of Infectious Diseases (Approval No. 1671, December 2025). All study participants provided informed consent.

## Conflicts of Interest

E.H. is employed by Niitaka Co. Ltd. However, this research was undertaken independently as part of a collaborative project between the Japan Institute for Health Security and Gunma Paz University. Niitaka Co. Ltd. did not participate in the study design; collection, analysis, and interpretation of data; report writing; and the decision to submit the paper for publication.

## Supporting information


**Figure S1:** Effect of ruxolitinib on ISG expression in GII.17 HuNoV‐infected J2 HIEs. J2 monolayers pretreated with DMSO (0.4%) or ruxolitinib (5 μM) were inoculated with a stool filtrate containing GII.17 (1.6 × 10^5^ GEs per well) in the presence of the specified compound and 500 μM GCDCA. At 1 and 48 hpi, total RNA was extracted and quantified using RT‐qPCR to determine relative *ISG* mRNA expression: (a) *ISG15*, (b) *RSAD2*, and (c) *ISG56*. Gene expression levels in DMSO‐treated cells at 1 hpi were set as 1. Data are presented as the mean ± SD (*n* ≥ 5). Statistical significance was assessed using the Mann–Whitney U test. Asterisks indicate statistically significant differences compared with the DMSO‐treated control at 48 hpi (*p < 0.05, **p < 0.01). hpi, h postinfection.
**Figure S2:** Cell‐derived virus stocks contain greater numbers of infectious progeny viruses than supernatant‐derived virus stocks in HIEs. J2 monolayers pretreated with ruxolitinib (5 μM) and TAK‐779 (30 μM) were inoculated with a stool filtrate containing GII.17 (1.6 × 10^5^ GEs per well) in the presence of the specified compounds and 500 μM GCDCA. At 96 hpi, supernatant‐ and cell‐derived virus stocks were prepared and used to inoculate newly prepared J2 monolayers as described in the Experimental Procedures. At 96 hpi, the cells and supernatants were collected, and viral RNA was quantified by RT‐qPCR. Data were obtained from a single independent experiment with two replicate wells per condition and are presented as the mean ± SD. hpi, h postinfection.
**Figure S3:** Replication of GII.17 P1 stocks generated with or without ruxolitinib and TAK‐779 during subsequent passaging in HIEs. J2 monolayers were inoculated with 4.0 × 10^4^ GEs per well of P1 stocks generated by DMSO or ruxolitinib (5 μM)/TAK‐779 (30 μM) treatment in a differentiation medium supplemented with the corresponding compounds and 500 μM GCDCA for 3 h at 37°C. Following infection, cells and culture supernatants were collected at 96 hpi, and viral RNA was quantified by RT‐qPCR. Data were obtained from a single independent experiment with six replicate wells per condition and are presented as the mean ± SD. hpi, h postinfection.

## Data Availability

The data that support the findings of this study are available from the corresponding author upon reasonable request.
