# Supplementary material for: Treatment With Ruxolitinib and TAK‐779 Enhances GII.17 Human Norovirus Replication and Enables Serial Passaging in Human Intestinal Enteroids
Source: Genes Cells. 2026 Aug 1;31(5):e70139. doi: 10.1111/gtc.70139 (PMC13428328; doi:10.1111/gtc.70139)
Supplement: Supplementary file 1 — Figure S1: Effect of ruxolitinib on ISG expression in GII.17 HuNoV‐infected J2 HIEs. J2 monolayers pretreated with DMSO (0.4%) or ruxolitinib (5 μM) were inoculated with a stool filtrate containing GII.17 (1.6 × 10^5^ GEs per well) in the presence of the specified compound and 500 μM GCDCA. At 1 and 48 hpi, total RNA was extracted and quantified using RT‐qPCR to determine relative ISG mRNA expression: (a) ISG15, (b) RSAD2, and (c) ISG56. Gene expression levels in DMSO‐treated cells at 1 hpi were set as 1. Data are presented as the mean ± SD (n ≥ 5). Statistical significance was assessed using the Mann–Whitney U test. Asterisks indicate statistically significant differences compared with the DMSO‐treated control at 48 hpi (*p < 0.05, **p < 0.01). hpi, h postinfection. Figure S2: Cell‐derived virus stocks contain greater numbers of infectious progeny viruses than supernatant‐derived virus stocks in HIEs. J2 monolayers pretreated with ruxolitinib (5 μM) and TAK‐779 (30 μM) were inoculated with a stool filtrate containing GII.17 (1.6 × 10^5^ GEs per well) in the presence of the specified compounds and 500 μM GCDCA. At 96 hpi, supernatant‐ and cell‐derived virus stocks were prepared and used to inoculate newly prepared J2 monolayers as described in the Experimental Procedures. At 96 hpi, the cells and supernatants were collected, and viral RNA was quantified by RT‐qPCR. Data were obtained from a single independent experiment with two replicate wells per condition and are presented as the mean ± SD. hpi, h postinfection. Figure S3: Replication of GII.17 P1 stocks generated with or without ruxolitinib and TAK‐779 during subsequent passaging in HIEs. J2 monolayers were inoculated with 4.0 × 10^4^ GEs per well of P1 stocks generated by DMSO or ruxolitinib (5 μM)/TAK‐779 (30 μM) treatment in a differentiation medium supplemented with the corresponding compounds and 500 μM GCDCA for 3 h at 37°C. Following infection, cells and culture supernatants were collected at 96 hpi, and viral [file GTC-31-0-s001.pptx]

## Slide 1
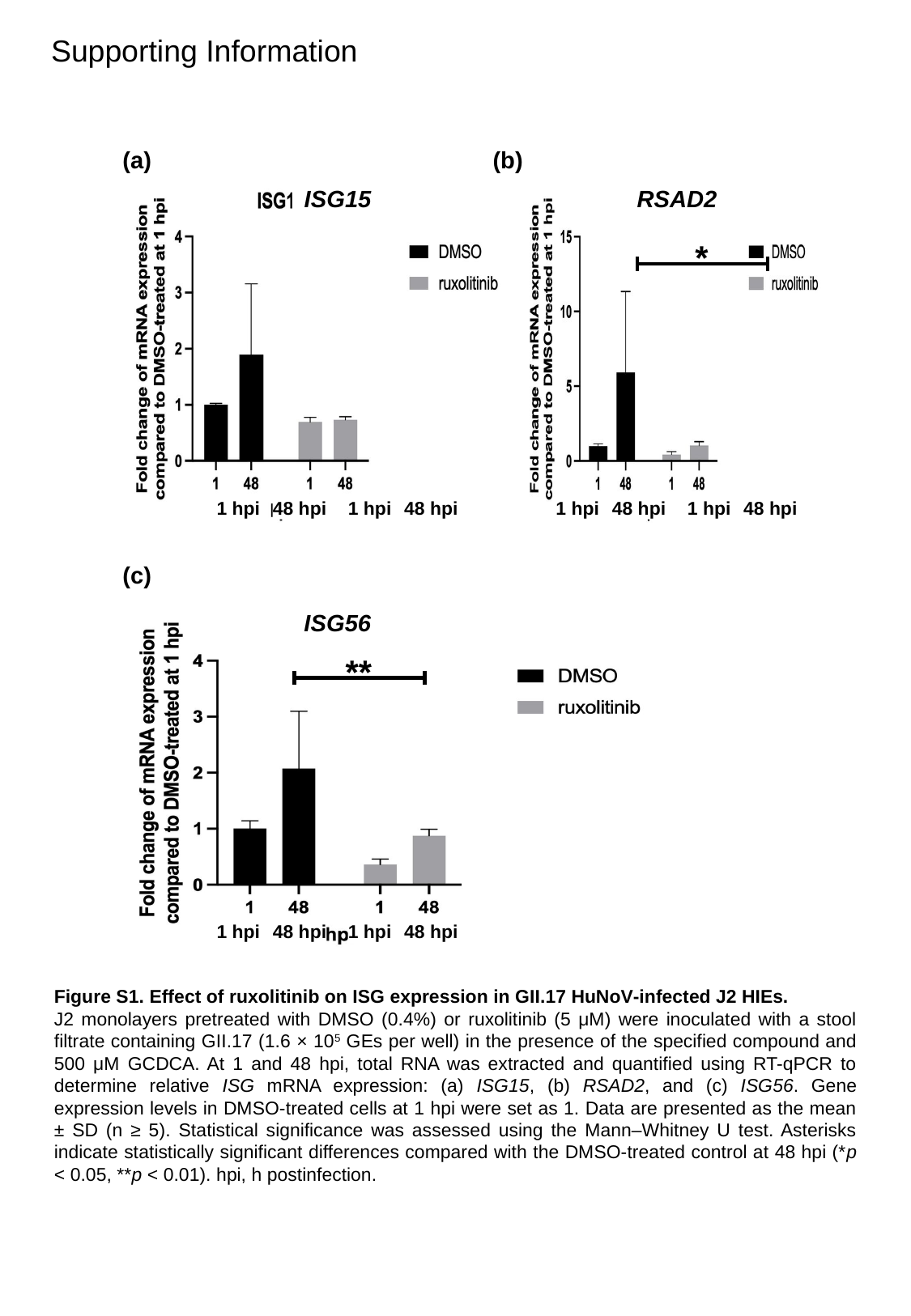

Supporting Information
(a)
(b)
ISG15
RSAD2
*
1 hpi
48 hpi
1 hpi
48 hpi
1 hpi
48 hpi
1 hpi
48 hpi
(c)
ISG56
**
1 hpi
48 hpi
1 hpi
48 hpi
Figure S1. Effect of ruxolitinib on ISG expression in GII.17 HuNoV‑infected J2 HIEs.
J2 monolayers pretreated with DMSO (0.4%) or ruxolitinib (5 μM) were inoculated with a stool filtrate containing GII.17 (1.6 × 10^5^ GEs per well) in the presence of the specified compound and 500 μM GCDCA. At 1 and 48 hpi, total RNA was extracted and quantified using RT-qPCR to determine relative ISG mRNA expression: (a) ISG15, (b) RSAD2, and (c) ISG56. Gene expression levels in DMSO-treated cells at 1 hpi were set as 1. Data are presented as the mean ± SD (n ≥ 5). Statistical significance was assessed using the Mann–Whitney U test. Asterisks indicate statistically significant differences compared with the DMSO-treated control at 48 hpi (*p < 0.05, **p < 0.01). hpi, h postinfection.

## Slide 2
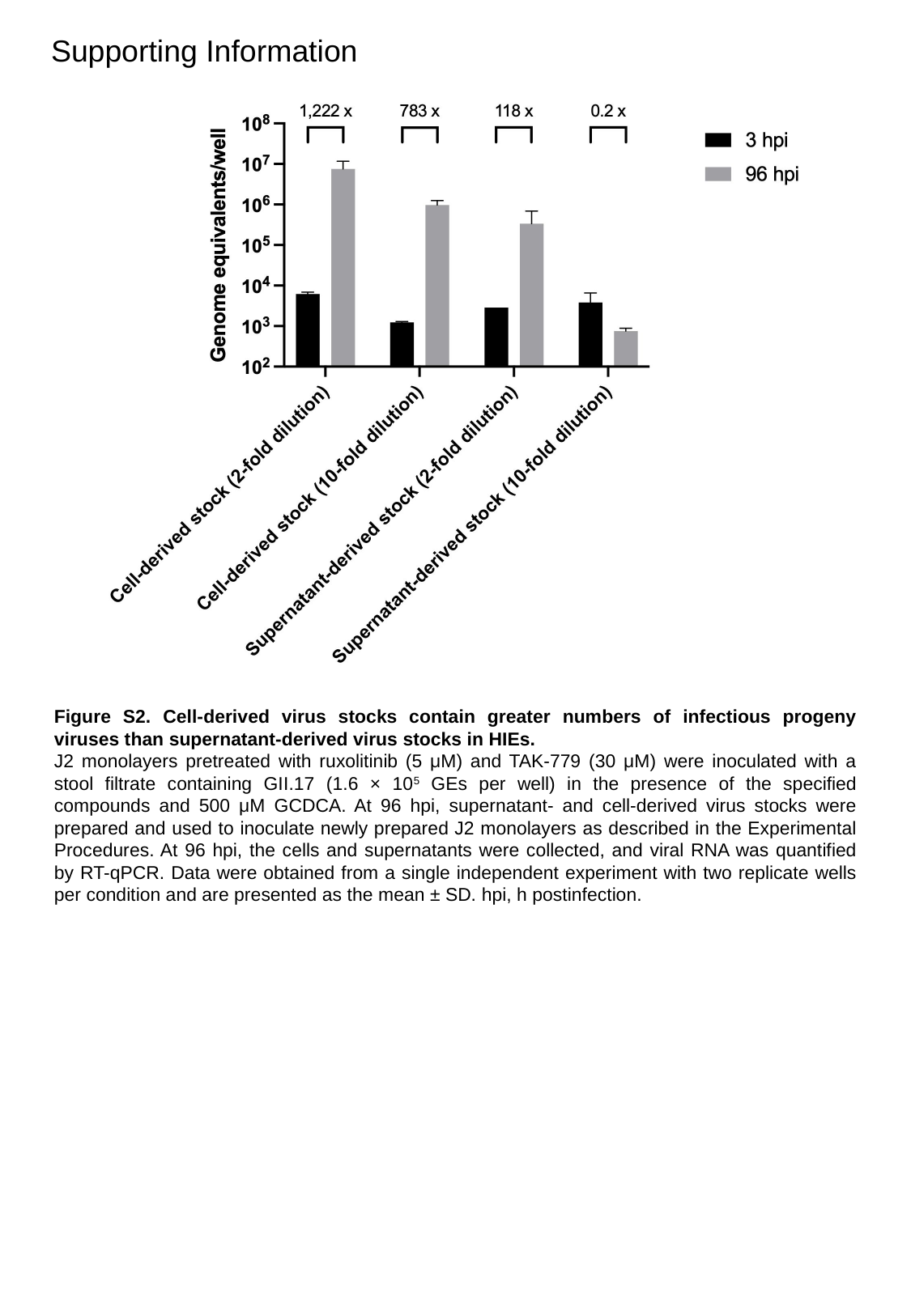

Supporting Information
Figure S2. Cell-derived virus stocks contain greater numbers of infectious progeny viruses than supernatant-derived virus stocks in HIEs.
J2 monolayers pretreated with ruxolitinib (5 μM) and TAK-779 (30 μM) were inoculated with a stool filtrate containing GII.17 (1.6 × 10^5^ GEs per well) in the presence of the specified compounds and 500 μM GCDCA. At 96 hpi, supernatant- and cell-derived virus stocks were prepared and used to inoculate newly prepared J2 monolayers as described in the Experimental Procedures. At 96 hpi, the cells and supernatants were collected, and viral RNA was quantified by RT-qPCR. Data were obtained from a single independent experiment with two replicate wells per condition and are presented as the mean ± SD. hpi, h postinfection.

## Slide 3
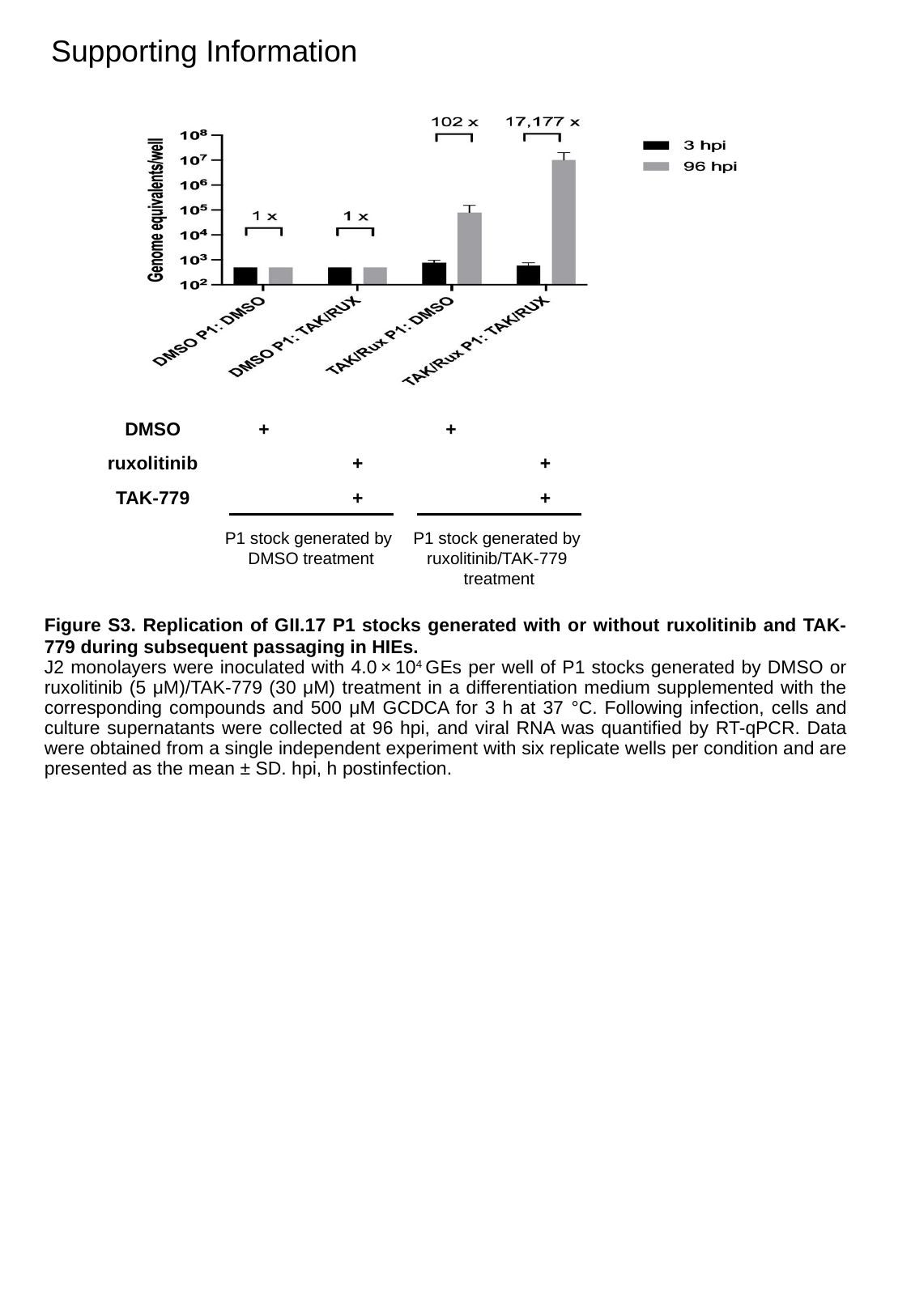

Supporting Information
DMSO
+
+
ruxolitinib
+
+
TAK-779
+
+
P1 stock generated by
DMSO treatment
P1 stock generated by
ruxolitinib/TAK-779
treatment
Figure S3. Replication of GII.17 P1 stocks generated with or without ruxolitinib and TAK-779 during subsequent passaging in HIEs.
J2 monolayers were inoculated with 4.0 × 10^4^ GEs per well of P1 stocks generated by DMSO or ruxolitinib (5 μM)/TAK-779 (30 μM) treatment in a differentiation medium supplemented with the corresponding compounds and 500 μM GCDCA for 3 h at 37 °C. Following infection, cells and culture supernatants were collected at 96 hpi, and viral RNA was quantified by RT-qPCR. Data were obtained from a single independent experiment with six replicate wells per condition and are presented as the mean ± SD. hpi, h postinfection.
